# Effect of water yam (*Dioscoreaalata*) flour fortified with distiller's spent grain on nutritional, chemical, and functional properties

**DOI:** 10.1002/fsn3.254

**Published:** 2015-07-03

**Authors:** Wasiu Awoyale, Busie Maziya‐Dixon, Lateef Oladimeji Sanni, Taofik Akinyemi Shittu

**Affiliations:** ^1^ International Institute of Tropical AgriculturePMB 5320 Oyo RoadIbadanOyo StateNigeria; ^2^ Department of Food, Agriculture and BioengineeringKwara State UniversityPMB 1530MaleteKwara StateNigeria; ^3^ Department of Food Science and TechnologyFederal University of Agriculture AbeokutaPMB 2240AbeokutaOgun StateNigeria

**Keywords:** Amino acids, dietary fiber, functional properties, nutrition, protein, Yam flour

## Abstract

It was envisaged that the inclusion of treated distiller's spent grain (DSG) to yam flour might increase its nutritional value, with the aim of reducing nutritional diseases in communities consuming yam as a staple. Hence, yam flour was fortified with DSG at 5–35%. The effects of this fortification on the nutritional, chemical, and functional properties of yam flour were investigated. The result showed a significant increase (*P* ≤ 0.001) in fat, ash, protein, total amino acids, total dietary fiber, and insoluble dietary fiber contents of the blends as DSG increased except for starch and soluble dietary fiber contents, which decreased. The functional properties showed a significant (*P* ≤ 0.001) reduction with DSG inclusion. The inclusion of DSG increased both the tryptophan and methionine contents of the blends. Therefore, the DSG fortified yam flour could contribute to quality protein intake in populations consuming yam as a staple, due to its indispensible amino acid content.

## Introduction

Yam is a staple crop in growing areas (Asiedu et al. 1992) with over 90% of the global production coming from West Africa with Nigeria as the leading producer (FAO, 2003). Yam is consumed in different ways, such as boiled, fried, or baked. Tubers are often dried and milled into flour for reconstituting into a stiff paste (*amala*), which is eaten with preferred vegetable soup (Awoyale et al. 2010). It is an elite crop and preferred over other crops in growing regions. It can be stored longer than other root and tuber crops, ensuring a food supply even at times of general scarcity. Yam is of major importance in the diet and economic life of people in West Africa, the Caribbean islands, Asia, and Oceania (Ravindran and Wanasundera 1992; Girardin et al. 1998). Information on the nutritive value of yam has been highlighted (Bradbury and Holloway 1988; Opara 1999; Alves 2000; Afoakwa and Sefa‐Dedeh 2001).

Yam tubers consist of about 21% dietary fiber and are rich in carbohydrates, vitamin C, and essential minerals. Worldwide annual consumption of yam is 18 million tons, with 15 million in West Africa. Annual consumption in West Africa is 61 kg per capita. Because of its perishability and bulkiness, it is processed into yam flour (Onwuka and Ihuma 2007). Yam flour is either in the fermented or unfermented form produced from white yam (*Dioscorea rotundata*) or water yam (*Dioscorea alata)*. However, because of the low protein content of *Dioscorea spp*. (Onayemi and Potter 1974), protein‐energy malnutrition is prevalent in rural populations where yam is a staple especially among women and children (Adamson 1985). Therefore, improving the nutritional quality of yam flour through fortification using a protein rich source will contribute in improving the nutrition status of women and children.

Fortification refers to the practice of deliberately increasing the content of essential nutrients in a food irrespective of whether the nutrients were originally in the food before processing or not, so as to improve the nutritional quality of the food supply and to provide a public health benefit with minimal risk to health and enrichment; which is defined as “synonymous with fortification refers to the addition of nutrients which are lost during processing to a food (FAO/WHO, 1994). The levels of food fortification depend on the nutritional needs of the population, amount consumed, and regulations in the country (Awoyale et al. 2010). Some research has been reported on the fortification of yam flour with different fortificants (Akingbala et al. 1996; Babajide et al. 2004; Abulude and Ojediran 2006). At present, there islimited information on the use of distillers' spent grain (DSG) to fortify yam flour.

Distiller's spent grain is a by‐product of cereal fermentation in the production of alcoholic beverages. It contains a high amount of proteins ranging from 23 to 35% and dietary fiber ranging from 27 to 55% (Rasco and McBurney 1989). It also contains yeast cell, vitamin B‐complexes, and other nutrients formed during the fermentation–distillation process (Kaiser 2006). The potential to utilize DSG products from wheat and other cereal grains as a high protein and fiber ingredient in formulated foods has received increasing attention (Morad et al. 1984; Wu et al. 1985; Rasco et al. 1987a,b). For instance, DSG has been added up to 35% by mass in brownies, chocolate chip, and spice and lemon molasses cookies, about 30 to 50% of yeast bread, 30% of quick breads to produce highly acceptable products (Rasco and McBurney 1989). In addition, Tsen et al. (1982) reported that chocolate chip cookies containing 15% dried distillers' grain residues were as acceptable as chocolate chip cookies containing no distillers' grain. However, no work has been reported on the effect of using DSG as a fortificant on staples such as yam flour commonly consumed in developing countries.

Therefore, this study was aimed at evaluating the effect of DSG fortification on the nutritional composition and functional properties of yam flour produced from water yam (*Dioscorea alata)*.

## Methods

The water yam variety TDd98/01166 was grown at the Research Farm of the International Institute of Tropical Agriculture (IITA), Ibadan, Nigeria and its tubers were used for the investigation. The DSG was obtained from United State Department of Agriculture‐Agricultural Research Service (USDA‐ARS), North Dakota, USA.

### Treatment of DSG

A suspension was made from 100 g of DSG containing alcohol and residual sugar in 400 mL of water and fermented with yeast (0.8 g) for 1 h to convert residual sugar to alcohol, which was then removed by distillation. The pH of the suspension was then adjusted to about 6.0 to 7.0 by adding sodium hydroxide (7 mL). The resulting suspension was dried to about 5–10% moisture content as reported by Awoyale et al. (2010).

### Production of yam flour

The yam tubers were peeled, washed, sliced into cubes, and dried in a hot air oven at 65°C for 48 h. The dried yam chips were milled into flour using an attrition mill, packaged in polyethylene bags, and stored until needed (Udensi et al. 2008).

### Formulation of DSG‐yam flour blends

Yam flour and DSG were weighed and mixed in ratios 100:0, 95:5, 90:10, 85:15, 80:20, 75:25, 70:30 and 65:35. Each mixture was thoroughly blended with a laboratory blender, packed and sealed in a low‐density polythene bag until required (Adelakun et al. 2004).

### Determination of chemical composition

The moisture, ash, and fat contents were determined using standard laboratory procedures (AOAC, 1990). The protein content was determined by Kjeldahl method using KjeltecTM model 2300 protein analyzer (Foss Analytical Manual, 2003). A conversion factor of 6.25 was used to convert total nitrogen to percent crude protein. The total sugar and starch contents were analyzed in duplicate, according to the method described by Dubois et al. (1956).

Samples were analyzed for soluble (SDF) and insoluble dietary fiber (IDF) fractions using the enzymatic‐gravimetric procedure (Prosky et al. 1988). Total dietary fiber (TDF) was calculated as the sum of SDF and IDF.

Amino acids were determined by reverse phase liquid chromatography (Waters 1500 Series HPLC; Milford, MA) with UV detection at 254 nm as reported by Cohen et al. (1988) at the South African Grain Laboratory, Pretoria, South Africa.

### Bulk density

Bulk density was determined using a standard laboratory method (AOAC, 1990). Flour samples were weighed (7 g) into a 50‐mL graduated measuring cylinder. The cylinder was tapped gently against the palm of the hand until a constant volume was obtained. Bulk density was calculated as weight of sample ⁄ volume of sample after tapping. All analyses were carried out in duplicates.

### Water absorption capacity and oil absorption capacity

Water absorption capacity (WAC) and oil absorption capacity (OAC) were determined using the method as described by Beuchat (1977). Flour sample (1 g) was mixed with 10 mL of distilled water for WAC and 10 mL of oil for OAC and blended for 30 sec. Each sample was allowed to stand for 30 min after which it was centrifuged at 1303 g for 30 min at room temperature. The supernatant was decanted. The weight of water or oil absorbed by the flour was calculated and expressed as percentage WAC or OAC.

### Swelling capacity

The method described by Ukpabi and Ndimele (1990) was used. Flour samples (10 g) were placed in a washed, dried, and weighed graduated measuring cylinder and 100 mL of distilled water was added. The suspension was stirred and allowed to stand for 1 h. The supernatant was discarded and the cylinder with its content weighed to obtain the weight of the net sample. Swelling capacity on volume basis was calculated as difference in final to initial volume of the sample.

### Least gelation concentration

The least gelation concentration was determined by the method described by Coffman and Garcia (1977). Ten suspensions (2, 4, 6, 8, 10, 12, 14, 16, 18, and 20% w/v) of the samples in 5‐mL distilled water were prepared in test tubes. The test tubes containing the suspensions were heated in a boiling water bath (Thelco, model 83, Missouri City, Texas, United States) for 1 h. The tubes and contents were cooled rapidly under running water and then cooled further for 2 h at 4°C. The tubes were then inverted to see if the contents would fall or slip off. The least gelation concentration is that concentration when the sample from the inverted test tube does not fall or slip off.

### Pasting properties

Pasting characteristics was determined using a Rapid Visco Analyzer (Model RVA‐4C, Newport Scientific, Warriewood, Australia) interfaced with a personal computer equipped with the Thermocline software supplied by the same manufacturer (Deffenbaugh and Walker 1989). A sample of 3 g (moisture content less than 12%) was weighed into a canister and made into slurry by adding 25 mL of distilled water. The canister (covered with a stirrer) was inserted into the RVA. The slurry was held at 50°C for 1 min, heated to 95°C within 3 min, and then held at 95°C for 2 min., cooled to 50°C within 3 min and then held at 50°C for 2 min, while maintaining a rotation speed of 160 rpm. The viscosity is expressed as Rapid Viscosity Units (RVU). Records of peak viscosity (the maximum viscosity during pasting), breakdown viscosity (the difference between the peak viscosity and the minimum viscosity during pasting), setback viscosity (the difference between the maximum viscosity during cooling and the minimum viscosity during pasting), final viscosity (the viscosity at the end of the RVA run), pasting temperature (^°^C) (the temperature at which there is a sharp increase in the viscosity of the flour suspension after the commencement of heating), and peak time (min) (time taken for the paste to reach the peak viscosity) were taken.

### Statistical analysis

Data were subjected to analysis of variance (ANOVA) using Statistical Analysis System (SAS) package (version 9.1, SAS Institute, Inc., Cary, NC) (SAS, 2003). Means were separated using Fischer's protected Least Significant Difference (LSD) test.

## Results

### Chemical composition

Presented in Table 1 is the chemical composition of the yam flour: DSG blends. Mean total ash content was 3.2% and ranged from 2.4% for 100% yam flour to 3.5% for the 90% yam: 10% DSG blends. There was a significant difference (*P* ≤ 0.001) in the ash content of the fortified yam flour. The fortified yam flour moisture content was also significantly (*P* ≤ 0.001) higher in 65% yam flour: 35% DSG blend (4.6%) and lower in 100% yam flour (3.6%) (Table 1). Furthermore, there was a significant (*P* ≤ 0.001) decrease in the starch content of the blends from 62% for 100% yam flour to 48% for 65% yam flour: 35% DSG blend (Table 1), showing that as the quantity of DSG increased, the starch content of the blends decreased. Additionally, there was a significant (*P* ≤ 0.001) increase in the fat content from 0.43% (100% yam flour) to 4.30% (65% yam flour: 35% DSG blend) (Table 1). Similarly, the protein content increased significantly (*P* ≤ 0.001) from 7.2% for 100% yam flour to 15.10% for 65% yam flour: 35% DSG blend (Table 1), revealing that for every increase in the quantity of DSG, there is a corresponding increase in the protein content of the blends.

**Table 1:** Chemical composition of yam flour (Y) and distillers' spent grain (DSG) blends

Samples	Ash (%)	Moisture (%)	Protein (%)	Sugar (%)	Starch (%)	Fat (%)	pH
100% DSG	2.37 ± 0.02^b^	5.06 ± 0.04^a^	29.80 ± 0.13^a^	3.37 ± 0.06^d^	23.86 ± 0.40^g^	9.63 ± 0.02^a^	5.70 ± 0.01^i^
100% Y	3.29 ± 0.02^c^	3.56 ± 0.08 ^g^	7.15 ± 0.31^i^	2.71 ± 0.10^e^	62.02 ± 0.11^a^	0.43 ± 0.00^i^	6.82 ± 0.00^a^
95% Y:5% DSG	3.33 ± 0.01^a^	3.62 ± 0.02 ^fg^	7.92 ± 0.10^h^	2.41 ± 0.03^f^	61.76 ± 0.63^a^	0.90 ± 0.02^h^	6.73 ± 0.00^b^
90% Y:10% DSG	3.35 ± 0.01^a^	3.68 ± 0.01^f^	9.15 ± 0.03^g^	2.82 ± 0.10^e^	61.40 ± 0.94^ab^	1.58 ± 0.07^g^	6.64 ± 0.01^c^
85% Y:15% DSG	3.30 ± 0.01^bc^	3.79 ± 0.04^e^	10.50 ± 0.07^f^	3.50 ± 0.18^d^	60.80 ± 0.12^b^	1.92 ± 0.05^f^	6.56 ± 0.00^d^
80% Y:20% DSG	3.34 ± 0.016^a^	3.94 ± 0.06^d^	11.55 ± 0.03^e^	3.58 ± 0.07^cd^	57.81 ± 0.22^c^	2.53 ± 0.12^e^	6.38 ± 0.01^e^
75% Y:25% DSG	3.33 ± 0.03^a^	4.20 ± 0.02^c^	12.76 ± 0.03^d^	3.71 ± 0.05^c^	53.03 ± 0.05^d^	3.79 ± 0.01^d^	6.33 ± 0.00^f^
70% Y:30% DSG	3.34 ± 0.02^a^	4.46 ± 0.04^b^	13.83 ± 0.01^c^	4.04 ± 0.06^b^	49.29 ± 0.51^e^	4.18 ± 0.00^c^	6.27 ± 0.01^g^
65% Y:35% DSG	3.32 ± 0.02^ab^	4.63 ± 0.01^a^	15.08 ± 0.10^b^	4.50 ± 0.18^a^	48.02 ± 0.42^f^	4.30 ± 0.08^b^	6.23 ± 0.00^h^
Mean	3.22	4.10	13.08	3.40	53.11	3.25	6.41
*P*‐level	a	a	a	a	a	a	a

a *P* ≤ 0.001. Means with different superscript along the same column are significantly different at *P* ≤ 0.05.

### Amino acids composition

The total amino acid composition of the fortified yam flour increased from 5.48 g/100 g for 100% yam flour to 13.91 g/100 g for the 65% yam flour: 35% DSG blend, with glutamic and aspartic acids contributing the highest percentage while tryptophan and methionine contributes the least (Table 2). It was observed that the amount of each amino acid in the blends increased as the quantity of DSG increased. Additionally, the inclusion of DSG increased both the quantity and quality of the protein in the yam flour (Table 2). The result also revealed that methionine and tryptophan were the limiting amino acids in the 100% yam flour. These amino acids were compensated for with the inclusion of DSG to the yam flour (Table 2).

**Table 2:** Amino acid composition (g/100 g sample) of yam flour (Y) and distillers' spent grains (DSG) blends

Amino Acids	100% DSG	100% Y	95% Y:5% DSG	90% Y:10% DSG	85% Y:15% DSG	80% Y:20% DSG	75% Y:25% DSG	70% Y:30% DSG	65% Y:35% DSG
Methionine	0.49	0.12	0.16	0.18	0.18	0.24	0.23	0.23	0.24
Aspartic acid	2.15	0.53	0.74	0.81	0.88	0.94	1.01	1.06	1.13
Glutamic acid	6.22	0.89	1.26	1.51	1.74	1.94	2.17	2.43	2.61
Serine	1.74	0.42	0.48	0.56	0.62	0.67	0.73	0.81	0.84
Glycine	1.24	0.25	0.31	0.36	0.41	0.45	0.50	0.56	0.59
Histidine	0.87	0.18	0.23	0.26	0.29	0.31	0.37	0.41	0.42
Arginine	1.34	0.47	0.50	0.54	0.58	0.63	0.67	0.79	0.88
Threonine	1.24	0.24	0.30	0.35	0.40	0.43	0.50	0.54	0.60
Alanine	2.45	0.30	0.41	0.53	0.62	0.71	0.80	0.92	0.99
Proline	2.79	0.27	0.40	0.56	0.66	0.76	0.89	1.02	1.13
Tyrosine	1.07	0.23	0.23	0.23	0.26	0.37	0.38	0.41	0.49
Valine	1.45	0.27	0.34	0.41	0.45	0.51	0.57	0.63	0.68
Isoleucine	1.09	0.23	0.29	0.34	0.36	0.41	0.45	0.51	0.53
Leucine	3.87	0.43	0.60	0.77	0.90	1.07	1.21	1.37	1.51
Phenylalanine	1.66	0.34	0.41	0.47	0.50	0.58	0.64	0.72	0.74
Lysine	0.82	0.24	0.25	0.26	0.27	0.33	0.35	0.41	0.41
Trypthophan	0.23	0.09	0.09	0.10	0.15	0.15	0.14	0.12	0.14
TAa	30.72	5.48	6.98	8.22	9.28	10.49	11.6	12.93	13.91
TIAa With Histidine	11.73	2.14	2.66	3.12	3.50	4.03	4.46	4.93	5.26
TIAa Without Histidine	10.85	1.96	2.43	2.86	3.20	3.71	4.09	4.53	4.83
TCIAa	2.42	0.69	0.73	0.77	0.84	0.99	1.05	1.20	1.37
TDAa	16.58	2.65	3.60	4.33	4.95	5.47	6.09	6.79	7.29

TIAa, total indispensable amino acids; TCIAa, total conditional indispensable amino acids; TDAa, total dispensable amino acids; TAa, total amino acid.

### Dietary fiber content

The total dietary fiber (TDF) content of the fortified yam flour increased from 6.02% for 100% yam flour to 11.7% for 65% yam flour: 35% DSG blend, out of which the insoluble dietary fiber (IDF) had the highest value (23.50%) and the soluble dietary fiber (SDF) the least (0.90%) (Table 3).

**Table 3:** Dietary fiber content of yam flour (Y) and distillers' spent grain (DSG) blends

Samples	Dietary fibers (%)		
	Insoluble	Soluble	Total
100% DSG	23.53	0.67	24.20
100% Y	5.10	0.92	6.02
95% Y:5% DSG	5.47	0.91	6.37
90% Y: 10% DSG	6.38	0.87	7.25
85% Y:15% DSG	8.27	0.85	9.13
80% Y:20% DSG	8.67	0.78	9.45
75% Y:25% DSG	9.59	0.76	10.34
70% Y:30% DSG	10.05	0.75	10.80
65% Y:35% DSG	11.01	0.72	11.73
Mean	9.79	0.80	10.59
Minimum	5.10	0.67	6.02
Maximum	23.53	0.92	24.20

### Functional properties

The functional properties of yam flour and DSG blend are presented in Table 4. It was observed that blending yam flour with DSG significantly (*P* ≤ 0.001) decreased oil absorption capacity (OAC) of 100% yam flour from 213 to 183% for 90% yam flour: 10% DSG blend (Table 4). There was a decrease in the water absorption capacity (WAC) from 260% for 100% yam flour to 241% for the 75% yam flour: 25% DSG blend (Table 4). Swelling capacity of the fortified yam flour similarly decreased from 3.40 for 100% yam flour to 2.40 for 65% yam flour: 35% DSG blend (Table 4). Significant differences (*P* ≤ 0.001) were observed for bulk density of the fortified yam flour; which decreased as the amount of added DSG increased. It ranged from 57% for 80% yam flour: 20% DSG blend to 70% for 100% yam flour (Table 4).

**Table 4:** Functional characteristics of yam flour (Y) and distillers' spent grain (DSG) blends

Samples	OAC (%)	WAC (%)	SWC	BD (%)	Amylose (%)	LGC (%)
100% DSG	216.00 ± 0.02^a^	291.00 ± 0.03^a^	2.27 ± 0.03^h^	58.00 ± 0.00^e^	3.18 ± 0.24^e^	20.10
100% Y	213.00 ± 0.13^a^	260.00 ± 0.03^b^	3.42 ± 0.02^a^	70.00 ± 0.00^a^	22.34 ± 0.15^a^	20.20
95% Y:5% DSG	191.00 ± 0.03^bc^	261.00 ± 0.02^b^	3.23 ± 0.05^b^	60.00 ± 0.01^d^	22.07 ± 0.24^a^	20.06
90% Y:10% DSG	183.00 ± 0.01^c^	243.00 ± 0.02^de^	3.09 ± 0.03^c^	63.00 ± 0.01^c^	19.83 ± 0.88^b^	20.02
85% Y:15% DSG	189.00 ± 0.03^bc^	245.00 ± 0.00^d^	2.72 ± 0.02^e^	66.00 ± 0.02^b^	17.77 ± 0.35^c^	20.04
80% Y:20% DSG	194.00 ± 0.01^b^	243.00 ± 0.01^de^	2.83 ± 0.06^d^	57.00 ± 0.01^e^	17.63 ± 0.38^c^	20.02
75% Y:25% DSG	193.00 ± 0.02^bc^	241.00 ± 0.01^d^	2.75 ± 0.02^e^	60.00 ± 0.01^d^	17.24 ± 0.65^c^	20.06
70% Y:30% DSG	191.00 ± 0.08^bc^	252.00 ± 0.02^c^	2.55 ± 0.02^f^	60.00 ± 0.01^d^	16.36 ± 0.12^d^	20.06
65% Y:35% DSG	186.00 ± 0.01^bc^	253.00 ± 0.01^c^	2.35 ± 0.02^g^	63.00 ± 0.01^c^	15.92 ± 0.03^d^	20.06
Mean	195.11	254.33	2.80	61.89	16.85	20.07
*P*‐level	a	a	a	a	a	

OAC‐Oil absorption capacity, WAC‐Water absorption capacity, SWC‐Swelling capacity, BD‐Bulk density, LGC‐Least gelation concentration.

a *P* ≤ 0.001. Means with different superscript along the same column are significantly different at *P* ≤ 0.05.

### Pasting properties

Table 5 showed the pasting properties of the yam flour and DSG blends. The peak viscosity, which is the highest viscosity of the range from 44RVU for 65% yam flour: 35% DSG blend to 198RVU for 100% yam flour. The values of the fortified yam flour final viscosity ranged between 63RVU for 65% yam flour: 35% DSG blend and 235RVU for 100% yam flour (Table 5). The setback viscosity also ranged from 26RVU for 65% yam flour: 35% DSG blend to 41RVU for 100% yam flour (Table 5). The result also revealed that the peak time ranged between 6 and 7 min and the pasting temperature ranged from 61.90°C for the 80% yam flour: 20% DSG blend to 62.30°C for the 75% yam flour: 25% DSG blend, respectively (Table 5).

**Table 5:** Pasting properties of yam flour (Y) and distillers' spent grain (DSG) blends

Samples	Peak (RVU)	Trough (RVU)	Break down (RVU)	FinalVisc. (RVU)	Set back (RVU)	Peak Time (min)	Pasting Temp (^°^C)
100% DSG	6.54 ± 0.54^i^	−11.50 ± 0.58^i^	18.04 ± 1.13^a^	−6.21 ± 3.71^i^	5.29 ± 4.29^e^	0.03 ± 0.00^d^	61.37 ± 0.39^b^
100% Y	198.46 ± 1.88^a^	194.38 ± 0.13^a^	4.08 ± 2.00^d^	235.25 ± 2.67^a^	40.88 ± 2.79^b^	6.53 ± 0.07^b^	61.90 ± 0.00^a^
95% Y:5% DSG	157.54 ± 2.04^b^	147.13 ± 1.96^b^	10.42 ± 0.08^b^	195.08 ± 2.58^b^	47.96 ± 0.63^a^	6.23 ± 0.10^c^	61.93 ± 0.03^a^
90% Y:10% DSG	139.46 ± 5.88^c^	135.67 ± 4.92^c^	3.79 ± 0.96^d^	176.92 ± 7.42^c^	41.25 ± 2.50^b^	6.23 ± 0.10^c^	61.93 ± 0.03^a^
85% Y:15% DSG	115.46 ± 2.88^d^	114.33 ± 3.17^d^	1.13 ± 0.29^f^	154.79 ± 2.46^d^	40.46 ± 0.71^b^	6.47 ± 0.13^b^	61.90 ± 0.05^a^
80% Y:20% DSG	97.67 ± 2.42^e^	95.67 ± 1.83^e^	2.00 ± 0.58^ef^	134.96 ± 3.71^e^	39.29 ± 1.88b^c^	6.90 ± 0.03^a^	61.88 ± 0.03^a^
75% Y:25% DSG	75.50 ± 0.75^f^	72.04 ± 0.54^f^	3.46 ± 1.29^de^	108.38 ± 0.63^f^	36.33 ± 0.08^c^	6.93 ± 0.07^a^	62.25 ± 0.50^a^
70% Y:30% DSG	57.88 ± 1.29^g^	50.67 ± 1.50^g^	7.21 ± 0.21^c^	79.42 ± 2.00^g^	28.75 ± 0.50^d^	7.00 ± 0.00^a^	61.90 ± 0.05^a^
65% Y:35% DSG	43.71 ± 1.46^h^	36.83 ± 1.75^h^	6.88 ± 0.29^c^	63.08 ± 1.08^h^	26.25 ± 0.67^d^	7.00 ± 0.00^a^	61.95 ± 0.05^a^
Mean	99.14	92.80	6.33	126.85	34.05	5.92	61.89
*P*‐level	a	a	a	a	a	a	NS

a *P* ≤ 0.001. Means with different superscript along the same column are significantly different at *P* ≤ 0.05.

## Discussion

### Chemical composition

Yam flour is a fermented or unfermented flour produced from either white yam (*Dioscorea rotundata)* or water yam (*Dioscorea alata*). However, because of its low protein content (Onayemi and Potter 1974), protein‐energy malnutrition is prevalent in rural populations where yam is consumed as a staple, especially among women and children (Adamson 1985). Hence, the reason for the fortification of yam flour with DSG.

The result of this investigation showed that the protein content of the flour blends increased as the quantity of DSG increased. This could be attributed to the high protein content of DSG (29.80%) compared to that of the 100% yam flour (7.15%). Similar results for the protein content of the 100% yam flour had been reported for the flour produced from different yam varieties (Bokanga 2000; Udensi et al. 2008; Alozie et al. 2009; Baah et al. 2009). Furthermore, Abulude and Ojediran (2006) reported comparable results with some of yam blends, for the protein contents of yam–cassava and yam–plantain blends.

The increase in fat content of the flour blends with DSG inclusion could be attributed to the high fat content of DSG. Comparable value (0.42%) with the fat content of the 100% yam flour was reported by Bradbury and Holloway (1988) and Souci et al. (1994). Additionally, the fat contents (0.04–2.00%) reported by Osagie (1992) for yam tubers were also comparable with that of the present study.

Ash content is a reflection of the mineral status, even though contamination can indicate a high concentration in a sample (Baah et al. 2009). The ash content of different varieties of *Dioscorea alata* flour reported by Lebot et al. (2005) and Baah et al. (2009) was in range with that of the 100% yam flour*,* while that of Osagie (1992) and Udensi et al. (2008) were lower compared to the results of this investigation.

The decrease in the starch content of the flour blends with corresponding increase in DSG inclusion may be attributed to the low starch content of DSG. This might have a negative effect on the acceptability of the reconstituted paste (*amala*), as starch is a very important factor for gel formation. However, similar observations with the starch content of the 100% water yam flour were made by other researchers (Maziya‐Dixon and Asiedu 2003; Lebot et al. 2005; Baah et al. 2009).

The moisture content of all the yam flour blends is still below the recommended safe level (12–13%) for storage of flour (FAO, 1992). This implied that all the blends might be stored for a long period before being used for the preparation of *amala*, without microbial contamination (Pierre 1989), if properly packaged in an airtight packaging material.

### Amino acid composition

Amino acid assay of foods is an important quality index, from which useful information on the nutritional quality and authenticity of food products and sources of raw materials used in food manufacture could be revealed (Alozie et al. 2009). The results obtained for the amino acids composition of the 100% yam flour was comparable with the observation made by Ekpeyong (1984) on yam tuber. However, it was observed that the inclusion of DSG to yam flour improves the amount of its limiting amino acids (methionine and tryptophan). This could be due to the level of these amino acids in DSG. In addition, FAO/WHO/UNN (1985) reported that the total indispensable amino acid requirements (g/100 g protein) are between 24.10 and 12.70 (with histidine) and between 22.20 and 11.10 (without histidine) for school children (10–12 years) and adults, respectively. Consequently, the consumption of the reconstituted paste (*amala*) produced from all the yam blends with total indispensable amino acid content (g/100 g protein) range of 28.09–34.34 (with histidine) and 25.89–31.72 (without histidine) by the target group (children and adults) might increase their total indispensible amino acids level required for growth and repair of worn‐out tissue.

### Dietary fiber content

Fiber is a type of carbohydrate that the body cannot digest. Though most carbohydrates are broken down into sugar molecules, fiber cannot be broken down into sugar molecules, and instead it passes through the body undigested. Fiber helps regulate the body's use of sugars, helping to keep hunger and blood sugar in check. Children and adults need at least 20 to 30 grams of fiber per day for good health. Great sources are whole fruits and vegetables, whole grains, and beans (Bauer and Turler‐Inderbitzin 2008). The values for the TDF content of the 100% yam flour in this study was similar to those reported by Baah et al. (2009) for different varieties of *Dioscorea alata*. Furthermore, Bauer and Türler‐Inderbitzin (2008), reported that decreased risk of coronary heart disease is correlated with increase in consumption of SDF and that, high water‐binding capacity of IDF results in the formation of softer stools which reduces the pressure necessary for the elimination of stools through the system faster, thus, less constipation and low incidence of maladies. Hence, *amala* produced from all the yam blends of this investigation might be able to suite this purpose when consumed by the target groups.

### Functional properties

As the functional properties of foods is known to affect the end use of any food and how such a food behaves during preparation for consumption, the functional properties of the fortified yam flour such as oil absorption capacity (OAC), water absorption capacity (WAC), swelling capacity (SWC), bulk density (BD), amylose content, and pasting properties would be of importance to the end users.

The OAC is important as oil acts as a flavor retainer and improves the mouth feel of foods (Kinsella 1976). The corresponding decrease in the OAC of the flour blends with DSG inclusion may be attributed to the low OAC of DSG. However, the OAC of this study was higher compared with that reported by Abulude and Ojediran (2006) on yam flour fortified with cassava and plantain flour. The WAC on the other hand, is the amount of water that an insoluble starch is able to hold in relation to its weight. High WAC is attributed to lose association of amylose‐amylopectin ratio in the native starch granule (Ayermor 1976). Similarly, the reduction in WAC of the flour blends may be due to the low WAC of the DSG, and reduction in amylose content of the blends as the proportion of DSG increased (Ayermor 1976). This result was comparable to the values (240–301%) reported by Abulude and Ojediran (2006) for the WAC of yam flour fortified with cassava and plantain flour.

Furthermore, the decrease in the SWC of the flour blends with increase in DSG may be due to the low starch content of DSG (Houssou and Ayernor 2002). Contrary to the observations made by Tester and Morrison (1990), the SWC of the flour blends increased as the amylose content increased.

Brennan et al. (1976) observed that high BD increases the rate of dispersion and as a result it is important in the reconstitution of yam *fufu* dough. This implied that 100% yam flour reconstituted into *amala* might be finer in texture compared to that of 80% yam flour: 20% DSG blend. In addition, 80% yam flour: 20% DSG blend might be easily packed for storage compared to that of 100% yam flour due to its low BD (Ikujenlola 2008).

The amylose content of the 100% yam flour of the present investigation was comparable to the work on *Dioscorea alata* and *Dioscorea rotundata*found in literature (Rasper and Coursey 1976; Bokanga 2000). The low starch content of DSG may be attributed to the low amylose content of the flour blends.

### Pasting properties

As the flour blends would be reconstituted to a thick paste (*amala*) before consumption, the pasting properties become important in predicting its behavior during and after cooking. Final viscosity is the most commonly used parameter to determine a particular starch‐based samples quality as it indicates the ability of the material to form gel after cooking (Sanni et al. 2006). The results showed that, the higher the proportion of DSG in the flour blend, the lower the final viscosity. This implied that, the 100% yam flour might quickly form a paste (*amala*) compared to the others due to its high final viscosity. Additionally, the higher the setback viscosity, the higher the rate of syneresis or weeping, and the easier it is for the food to be digested (Shittu et al. 2001). This means that a*mala* produced from 65% yam flour: 35% DSG might keep longer before “weeping” and as well digest fast when consumed due to its low setback value compared with that of 100% yam flour (Shittu et al. 2001). However, all the flour blends might be cooked to *amala* at a temperature of <63°C and time of <8 min., this implied low energy cost (Fasasi et al. 2007).

## Conclusion

The fat, ash, protein, sugar, total amino acids composition, total dietary fiber, and insoluble dietary fiber contents of the fortified yam flour increased while the starch and the soluble dietary fiber contents decreased as the DSG increased. In addition, aspertic and glutamic acids contributed the highest percentage of amino acids while tryptophan and methionine contributed the least in the flour blends. However, the inclusion of DSG to the yam flour increased both the tryptophan and methionine contents. There was also a decrease in the peak, final, and setback viscosities of the fortified yam flour. The WAC, OAC, swelling capacity, bulk density, and amylose content of the flour blends decreased with DSG inclusion. However, the DSG fortified yam flour could serve as a quality protein source when prepared to *amala* and consumed with preferred soup due to its high indispensible amino acid content.

## Conflict of Interest

None declared.
